# Dr. Clarence V. Gray

**Published:** 1916-08

**Authors:** 


**Dr. Clarence V. Gray,** Buffalo 1887, died at his home in Batavia, July 6, after an illness of two weeks. He practiced medicine in Elba for 20 years and then took charge of the Primrose Sanitarium which he has since conducted.

